# Seroprevalence of *Bartonella *in Eastern China and analysis of risk factors

**DOI:** 10.1186/1471-2334-10-121

**Published:** 2010-05-20

**Authors:** Jimin Sun, Guiming Fu, Junfen Lin, Xiuping Song, Liang Lu, Qiyong Liu

**Affiliations:** 1 Department of Vector Biology and Control,State Key Laboratory for Infectious Diseases Prevention and Control, National Institute for Communicable Disease Control and Prevention, Chinese Center for Disease Control and Prevention, Beijing 102206, China; 2 Zhejiang Provincial Center for Disease Control and Prevention, Hangzhou 310051, China

## Abstract

**Background:**

*Bartonella *infections are emerging in the Zhejiang Province of China. However, there has been no effort to date to explore the epidemiology of these infections in this region, nor to identify risk factors associated with exposure to *Bartonella*. The aim of this study was to investigate the seroprevalence of *Bartonella *in both patients bitten by dogs and blood donors (for control) in Eastern China, and to identify risk factors associated with exposure to *Bartonella*. As no previous data for this region have been published, this study will provide baseline data useful for *Bartonella *infection surveillance, control, and prevention.

**Methods:**

Blood samples were collected from industrial rabies clinic attendees and blood donors living in eight areas of the Zhejiang Province of China, between December 2005 and November 2006. An indirect immunofluorescent antibody test was used to determine the presence of *Bartonella *in these samples. Risk factors associated with *Bartonella *exposure were explored using Chi-square tests and logistic regression analysis of epidemiological data relating to the study's participants.

**Results:**

*Bartonella *antibodies were detected in 19.60% (109/556) of blood samples. Seroprevalence varied among the eight areas surveys, ranging from over 32% in Hangzhou to only 2% in Jiangshan (X^2 ^= 28.22, P < 0.001). We detected a significantly higher prevalence of *Bartonella *antibodies in people who had been bitten by dogs than in blood donors (X^2 ^= 13.86, P < 0.001). Seroprevalence of *Bartonella *was similar among males (18.61%, n = 317) and females (20.92%, n = 239).

**Conclusions:**

*Bartonella *antibodies were encountered in people living across Zhejiang Province and the seropositivity rate among those exposed to dog bites was significantly higher than that among blood donors, indicating that dog bites may be a risk factor for *Bartonella *infection.

## Background

The genus *Bartonella *comprises of fastidious, Gram-negative hemotropic bacteria that infect blood erythrocytes and endothelial cells of their hosts [1]. More than 20 species or subspecies of *Bartonella *have been described, of which at least 12 are recognized as human pathogens [2]. These species exploit a variety of different mammals as reservoir hosts and arthropods as vectors, and provoke a broad spectrum of manifestations in humans.

According to clinical reports, the number of patients with *Bartonella *infections in Zhejiang Province is the second largest in China. However, to date, there have been no efforts to explore the epidemiology of these infections in this region, nor to identify risk factors associated with exposure to *Bartonella*. Directly detecting *Bartonella *in humans is problematic, but detection of antibodies as an indication of exposure to the agent has been widely used in previous studies. In this study, we adopted the latter approach for the first time to investigate human exposure to *Bartonella *in the Zhejiang Province of China.

## Methods

### Blood samples

Patient blood samples (n = 205 from rabies clinics; n = 351 from normal blood donors) were collected from eight locations (Hangzhou, Tiantai, Longyou, Huzhou, Jiangshan, Chun'an, Longyou, and Linhai) of the Zhejiang Province of China from December 2005 to November 2006. The aims of our study were explained to all participants upon enrollment, and their consent was obtained prior to inclusion in this study. All enrolled participants provided information upon inclusion in the study, with regard to their age, gender, place of residence, and whether they had been exposed to dog bites.

Experimental research reported in this study has been performed with the approval of the ethics committee of Zhejiang Provincial Center for Disease Control and Prevention. Human research was carried out in compliance with the Helsinki Declaration.

### Serological testing

A commercially available *Bartonella henselae (B. henselae)*-based indirect immunofluorescence antibody (IFA) test kit (Euroimmun, Order No. FI219b-1005G) was used to assess the presence of IgG antibodies against *B. henselae *in human samples. After the samples were diluted 1:100, 1:320, 1:1000, 1:3200, and 1:10000 in phosphate-buffered saline PBS-Tween buffer (provided in the test kit), the IFA assay was conducted following the manufacturer's protocol. Positive and negative controls (provided in the test kit) were also used. Immunofluorescence was observed using an epifluorescence microscope at magnifications of 40× and 200×. According to the manufacturer's guidelines, a titer of 1:320 was considered indicative of an infection, and IgG antibody titers of 1:1000 or higher were judged as an indicator for acute infection.

### Data analysis

Chi-square tests and logistic regression analysis of *Bartonella *seroprevalence among sites, gender, age groups, and exposure to dog bites were conducted using the SPPS, version 11.0 statistical package (Chicago, IL, USA). The dependent variable in the logistic regression was assigned as the serological status of patients and the independent variables were site, gender, age, exposure, and site × exposure. The method of logistic regression used was forward-conditional. The stepwise probability was set to 0.05 for entry and 0.10 for removal. the classification cut-off was 0.5 and the maximum number of iterations was 20. Omnibus tests of model coefficients were also conducted.

## Results

Table 1 summarizes the results of positive antibody reactions in patients at various dilution titers of their blood samples. The proportion of titers of 1:1000 or higher among people bitten by dogs was consistently higher than those among healthy people who had not experienced bites. Furthermore, according to the manufacturer's instructions for data analysis, the final resultant mean of the proportion of acute infection in people exposed to dog bites was higher than that in control group.

**Table 1:** Results of positive reaction at different dilution titers

	Dilution titers				
	
	1:320	1:1000	1:3200	1:10000	Total
Bitten by dogs (n)	30	18	6	3	57
Control group (n)	43	8	1	0	52

Total (n)	73	26	7	3	109

Blood samples were collected from 556 people living in eight locations across Zhejiang Province (Table 2). Of these, 317 patients were male and 239 were female. Overall, 19.60% (109/556) of blood samples were seropositive for *Bartonella *based on antibody immunofluorescence results. Of note, seroprevalence of *Bartonella *varied significantly among sites within the Zhejiang Province (2.00-32.38%; X^2 ^= 28.220, P < 0.001]), and between those patients bitten by dogs versus those not bitten (27.80% and 14.81%, respectively; X^2 ^= 13.856, P < 0.001). Seroprevalence of *Bartonella *was found to be similar among males (18.61%, n = 317) and females (20.92%, n = 239; X^2 ^= 0.461, P = 0.497 > 0.05). Furthermore, participants were divided into four groups according to age and the seroprevalence in each was determined. In summary, we found that 17.78% (5/18) of those patients less than 15 years in age had *Bartonella *antibody, 18.27% (59/323) of patients between 15 and 44 years in age had antibodies, 18.42% (28/152) of patients between 45 and 59 years old had antibodies, and 26.98% (17/63) of those patients over 59 years in age had antibodies. These results suggest that age was an insignificant factor in *Bartonella *antibody expression (X^2 ^= 6.364, P > 0.05).

**Table 2:** Zhejiang Province site locations and seroprevalence incidence.

Site	Latitude	Longitude	Male (n)	Female (n)	Total (n)	*Bartonella *Positive (n)	Positive rate (%)
Hangzhou	30.15°N	120.20°E	59	46	105	34	32.38
Tiantai	29.14°N	121.03°E	51	53	104	27	25.96
Longyou	29.04°N	119.19E	30	20	50	11	22.00
Huzhou	20.52°N	120.04°E	34	18	52	8	15.38
Jiangshan	28.73°N	118.61°E	30	20	50	1	2.00
Chun'an	29.61°N	118.84°E	47	40	87	14	16.09
Longquan	28.09°N	119.12°E	31	21	52	8	15.38
Linhai	28.86°N	121.12°E	35	21	56	6	10.71

Total			317	239	556	109	19.60

The Chi-square value in omnibus tests of model coefficients was determined to be 60.326 (P < 0.05). Furthermore, the overall correct percentage was found to be 81.5%. Variables in the equation included site, exposure, and site×exposure. The wald of site×exposure, site, and exposure were determined to be 25.477 (P = 0.001), 20.090 (P = 0.005), and 16.174 (P = 0.024), respectively.

## Discussion

Various *Bartonella *species, including *B. henselae*, have previously been detected in domestic cats [3], small mammals, and *Haemaphysalis longicornis *and *Ixodes sinensis *ticks [4] in the Zhejiang Province of China. Of note, transmisson of *Bartonella*-related infection from any of these sources to humans is feasible. Therefore, it is necessary to study the prevalence of *Bartonella *in people throughout the Zhejiang Province and to analyze relevant risk factors of infection. In our study, the seroprevalence of *Bartonella *was found to be 14.81% among healthy patients, a percentage similar to the recently reported percentage (14.28%) recorded in the Yunnan Province in Southwestern China [5], but much lower than the percentage reported in Beijing (34.5%) [6]. Furthermore, Kikuchi *et al. *[7] previously reported a *B. henselae *specific IgG seroprevalence of 3.1% among adult patients with cardiovascular disease and 10.9% in a high-risk population of healthy veterinary students in Japan, of whom only 0.8% had positive results of serologic testing for *B. henselae *specific IgM. The bases for these differences are not clear, but are likely to be multi-factorial.

Due to the inherent serological cross-reactivity among *Bartonella *species in IFA assays [8,9], the observed seropositivity to *B. henselae *antigen may actually represent a previous or current infection with another *Bartonella *species. According to our analysis (carried out according to the manufacturer's instruction), cross reactivity with other *Bartonella *species cannot be ruled out; however, this kit has more than 84% specificity and 88% sensitivity for *B. henselae *IgG. As a result, our data suggest that detected antibodies were against all *Bartonella *species, while the majority of them were against *B. henselae*.

The fact that patients with *Bartonella *antibodies were detected across all eight study sites of the Zhejiang Province indicates that the general population in this region is at risk of exposure to *Bartonella*. Of note, the prevalence varied between sites within the province, although Hangzhou, Tiantai, and Longyou had the highest prevalence of positive reactions. Furthermore, the exposure rate due to dog bites, which was identified as a significant determinant of seroprevalence, also varied between sites, and was significantly higher in Hangzhou than other sites, and significantly lower in Jiangshan than other sites. Moreover, site×exposure fit the equation in logistic regression analysis, indicating that site and exposure results correlated to each other.

We also found that seroprevalence of *Bartonella *in individuals bitten by dogs was significantly higher than that among unbitten blood donors. However, the explanation of this finding is not altogether obvious, as domestic cats, rather than dogs, are considered a primary reservoir host for *Bartonella*. It is established that dogs serve as hosts for *Ctenocephalides felis*, the suspected vector of *Bartonella*. Therefore, dogs may acquire and maintain infected and infectious fleas, thereby, indirectly acting as a source of infection. Alternatively, people living in proximity to dogs may also be more likely to live in proximity to cats. However, it remains a possibility that dog bites themselves may transmit *Bartonella *infection, as *Bartonella *DNA has been detected in dog saliva [10].

Previously published studies have indicated that *B. henselae *infections are more common in children than adults. However, we did not see a significantly higher seropositivity among children than adults in our studies. In contrast, in our study the highest seropositivity was observed in the oldest (>59 years) patient age group. Others have reported similar findings [11].

## Conclusions

*Bartonella *infections can be difficult to diagnose clinically, particularly as many of the clinical presentations associated with them have nonspecific symptoms, such as fever. Thus, reliance on clinical reports from physicians to determine the public health burden of infection is not feasible. In contrast, serological surveys of individuals offer a means of quantifying the level of exposure to *Bartonella *among the population. In this study, we used this approach to demonstrate that exposure to this pathogen is both common and widespread across the Zhejiang Province of China, although prevalence was higher in some parts of the province than others. Furthermore, it appeared that people bitten by dogs were significantly more likely to have *Bartonella *antibodies than blood donors without exposure to bites. This information should serve to help those in relevant settings to consider *Bartonella *infection in the differential diagnosis of their patients.

## Competing interests

The authors declare that they have no competing interests.

## Authors' contributions

JS designed the study and drafted the manuscript. GF carried out the patient blood sample collection. JL performed analysis and interpretation of data. XS carried out serological testing. LL participated in the design of the study and helped draft the manuscript. QL conceived of the study and participated in its design and coordination. All authors read and approved the final manuscript.

## Pre-publication history

The pre-publication history for this paper can be accessed here:

http://www.biomedcentral.com/1471-2334/10/121/prepub
